# Inhibitory Effects of Surface Pre-Reacted Glass Ionomer Filler Eluate on *Streptococcus mutans* in the Presence of Sucrose

**DOI:** 10.3390/ijms25179541

**Published:** 2024-09-02

**Authors:** Mariko Kametani, Tatsuya Akitomo, Masakazu Hamada, Momoko Usuda, Ami Kaneki, Masashi Ogawa, Shunya Ikeda, Yuya Ito, Shuma Hamaguchi, Satoru Kusaka, Yuria Asao, Yuko Iwamoto, Chieko Mitsuhata, Yuto Suehiro, Rena Okawa, Kazuhiko Nakano, Ryota Nomura

**Affiliations:** 1 Department of Pediatric Dentistry, Graduate School of Biomedical and Health Sciences, Hiroshima University, Hiroshima 734-8553, Japan; mrysk25@hiroshima-u.ac.jp (M.K.); takitomo@hiroshima-u.ac.jp (T.A.); musuda@hiroshima-u.ac.jp (M.U.); kaneki@hiroshima-u.ac.jp (A.K.); caries0@hiroshima-u.ac.jp (M.O.); shunyaikeda@hiroshima-u.ac.jp (S.I.); yuuya@hiroshima-u.ac.jp (Y.I.); syuumai@hiroshima-u.ac.jp (S.H.); higech@hiroshima-u.ac.jp (S.K.); yuriaasao@hiroshima-u.ac.jp (Y.A.); yuko-tulip@hiroshima-u.ac.jp (Y.I.); chiekom@hiroshima-u.ac.jp (C.M.); 2 Department of Oral & Maxillofacial Oncology and Surgery, Osaka University Graduate School of Dentistry, Suita 565-0871, Japan; hamada.masakazu.dent@osaka-u.ac.jp; 3 Department of Pediatric Dentistry, Osaka University Graduate School of Dentistry, Suita 565-0871, Japan; suehiro.yuto.dent@osaka-u.ac.jp (Y.S.); okawa.rena.dent@osaka-u.ac.jp (R.O.); nakano.kazuhiko.dent@osaka-u.ac.jp (K.N.)

**Keywords:** surface pre-reacted glass ionomer (S-PRG) filler, *Streptococcus mutans*, ions, sucrose, RNA sequence

## Abstract

The surface pre-reacted glass ionomer (S-PRG) filler is a type of bioactive functional glass that releases six different ions. This study examined the effects of the S-PRG filler eluate on *Streptococcus mutans* in the presence of sucrose. In a solution containing *S. mutans*, the concentrations of BO_3_^3−^, Al^3+^, Sr^2+^, and F^−^ were significantly higher in the presence of the S-PRG filler eluate than in its absence (*p* < 0.001). The concentrations of these ions further increased in the presence of sucrose. Additionally, the S-PRG filler eluate significantly reduced glucan formation by *S. mutans* (*p* < 0.001) and significantly increased the pH of the bacterial suspension (*p* < 0.001). Bioinformatic analyses revealed that the S-PRG filler eluate downregulated genes involved in purine biosynthesis (purC, purF, purL, purM, and purN) and upregulated genes involved in osmotic pressure (opuAa and opuAb). At a low pH (5.0), the S-PRG filler eluate completely inhibited the growth of *S. mutans* in the presence of sucrose and significantly increased the osmotic pressure of the bacterial suspension compared with the control (*p* < 0.001). These findings suggest that ions released from the S-PRG filler induce gene expression changes and exert an inhibitory effect on *S. mutans* in the presence of sucrose.

## 1. Introduction

More than 700 species of bacteria can inhabit the human oral cavity [1]; of these, 100–200 are typically found in a single individual [2]. *Streptococcus* species are the most abundant, comprising approximately 15%–60% of all oral bacteria [3,4]. Bacteria in the oral cavity interact with a thin layer of enamel pellicle derived from salivary proteins to form biofilms on tooth surfaces [5]. *Streptococcus* and *Actinomyces* are early colonizers in biofilm formation, and subsequent environmental changes lead to increased bacterial diversity [6].

The consumption of fermentable sugars can disrupt biofilm homeostasis. Bacteria produce lactic acid from these sugars, leading to the emergence of acid-adapted species [7,8,9]. These acid-adapted bacteria gain a selective advantage and contribute to the development of dental caries [8,9]. In particular, *Streptococcus mutans* thrives in acidic environments and promotes the progression of severe caries, leading to the demineralization of tooth surfaces [10].

A surface pre-reacted glass ionomer (S-PRG) filler is a bioactive functional glass composed of a three-layer structure. A glass ionomer phase is formed on the surface of a multifunctional glass filler and then coated with a surface modification layer [11]. The S-PRG filler releases six types of ions from the glass ionomer phase: borate (BO_3_^3−^), aluminum (Al^3+^), silicate (SiO_3_^2−^), strontium (Sr^2+^), sodium (Na^+^), and fluoride (F^−^) [12,13]. The S-PRG filler exhibits various activities that help prevent dental caries, such as acid neutralization, the strengthening of tooth structure, and inhibition of dental plaque formation [14,15,16]. Accordingly, the S-PRG filler is widely used in dental materials such as composite resins, bonding agents, resin sealants, toothbrushes, and professional mechanical tooth cleaning (PMTC) pastes [12,17,18,19].

Several studies have shown that the S-PRG filler inhibits *S. mutans* biofilm formation in the presence of sucrose, a key factor in the development of dental caries [11,20,21]. However, few studies have thoroughly investigated the effect of the S-PRG filler on *S. mutans* beyond the simple inhibition of biofilm formation. The present study analyzed the effects of S-PRG eluate on the cariogenic activity of *S. mutans* in the presence of sucrose. We focused on the concentration of ions surrounding *S. mutans*, the comprehensive analysis of bacterial gene expression, and the resulting inhibitory effect due to gene expression changes.

## 2. Results

### 2.1. Ion Release from S-PRG Filler Eluate Surrounding S. mutans

**Figure 1:**
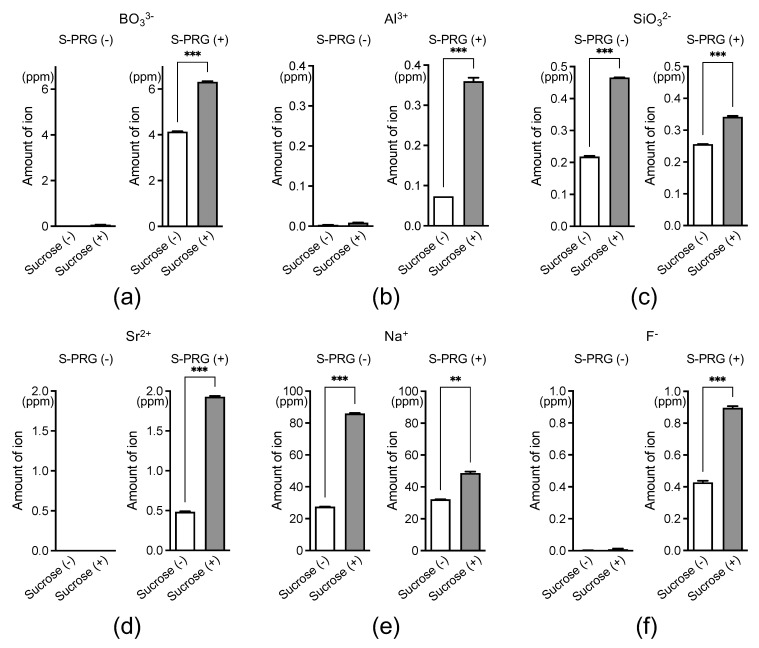
Ion concentrations around *S. mutans* derived from the S-PRG filler eluate, with or without sucrose. BO_3_^3−^ (**a**), Al^3+^ (**b**), SiO_3_^2−^ (**c**), Sr^2+^ (**d**), Na^+^ (**e**), and F^−^ (**f**). Data are presented as means ± standard deviations. ** *p* < 0.01, *** *p* < 0.001 between groups.

We first cultured *S. mutans* with an S-PRG filler eluate, with or without sucrose, and measured the concentration of ions released from the eluate surrounding *S. mutans*. Of the six ions released from the S-PRG filler eluate, BO_3_^3−^, Al^3+^, Sr^2+^, and F^−^ were detected at higher concentrations in the presence of the eluate, regardless of the presence or absence of sucrose (Figure 1). Notably, the concentration of these four ions surrounding *S. mutans* was significantly higher in the presence of sucrose than in the absence of the S-PRG filler eluate (*p* < 0.001).

### 2.2. Effect of S-PRG Filler Eluate on Glucan Formation and pH in S. mutans Cultures

**Figure 2:**
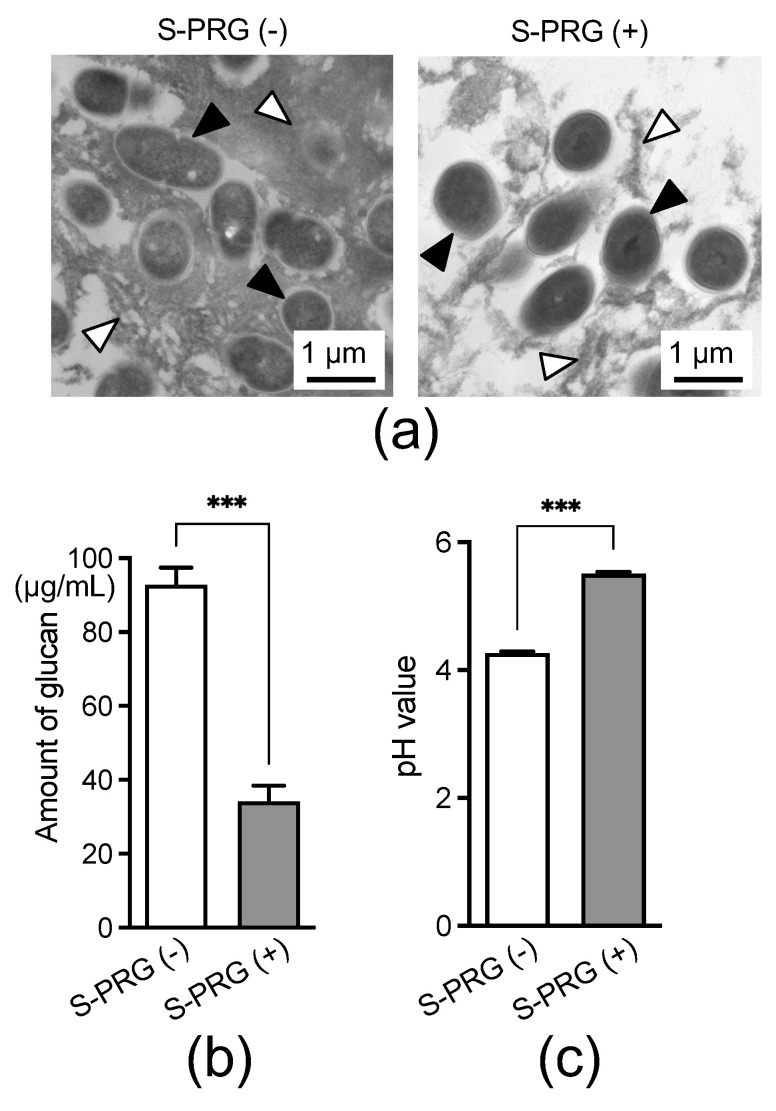
The effect of S-PRG filler eluates on glucan synthesis by *S. mutans* in the presence of sucrose and on the pH of the bacterial culture. (**a**) Transmission electron microscopy (TEM) images of *S. mutans* in the presence of 1% sucrose. Black arrowheads indicate *S. mutans* and white arrowheads indicate glucan. (**b**) Glucan levels in *S. mutans* cultures with 1% sucrose. (**c**) pH in *S. mutans* cultures with 1% sucrose. Data are presented as means ± standard deviations. *** *p* < 0.001 between groups.

Considering that several ions derived from the S-PRG filler eluate were prominently detected around *S. mutans* in the presence of sucrose, we analyzed the effects of the S-PRG filler eluate on the glucan synthesis ability of *S. mutans* in the presence of sucrose; we also evaluated the effects of the eluate on the pH of the bacterial culture. Transmission electron microscopy (TEM) images showed that the presence of S-PRG filler eluates reduced glucan formation around *S. mutans* cultured with 1% sucrose (Figure 2a). The amount of glucan synthesized by *S. mutans* was significantly lower in the presence of the S-PRG filler eluate than in its absence (*p* < 0.001) (Figure 2b). Additionally, the pH of the *S. mutans* culture was significantly higher in the presence of the S-PRG filler eluate than in its absence (*p* < 0.001) (Figure 2c).

### 2.3. S-PRG Filler Eluate Affects S. mutans Gene Expression in the Presence of Sucrose

**Figure 3:**
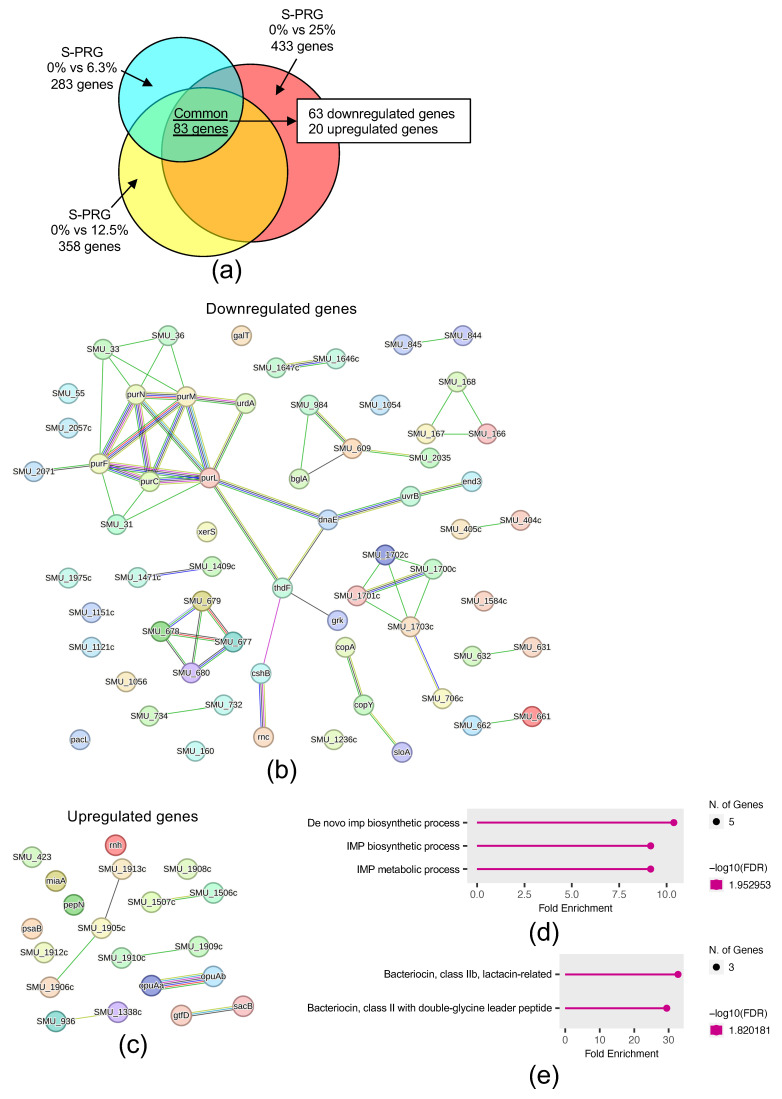
Comprehensive analysis of gene expression changes in *S. mutans* exposed to S-PRG filler eluates in the presence of sucrose. (**a**) A number of *S. mutans* genes with ≥2-fold expression changes in the presence of 1% sucrose and 6.3%, 12.5%, or 25% S-PRG filler eluate, compared with no S-PRG filler eluate, as determined by microarray analysis. Protein–protein interaction (PPI) networks of 63 downregulated genes (**b**) and 20 upregulated genes (**c**) were observed when *S. mutans* was treated with 6.3%, 12.5%, or 25% S-PRG filler eluate in the presence of 1% sucrose compared with the absence of the eluate. GO enrichment analysis of biological processes using ShinyGO for 63 downregulated genes (**d**) and 20 upregulated genes (**e**).

To identify genes affected by the S-PRG filler eluate, we performed a microarray assay on *S. mutans* cultured with 1% sucrose. The microarray analysis compared three conditions with varying S-PRG filler eluate concentrations: 0% versus 6.3%, 0% versus 12.5%, and 0% versus 25.0%. Under all conditions, 83 genes exhibited a ≥2-fold change in expression in the presence of the S-PRG filler eluate compared with its absence (Figure 3a). Of these 83 genes, 63 were downregulated, and 20 were upregulated in the presence of the eluate. Protein–protein interaction (PPI) network analysis revealed strong networks for the downregulated genes involved in purine biosynthesis (purC, purF, purL, purM, and purN) (Figure 3b) and the upregulated genes involved in osmotic stress response (opuAa and opuAb) (Figure 3c). Gene Ontology (GO) enrichment analysis showed that the 63 downregulated genes were associated with inosine monophosphate (IMP) biosynthesis and metabolism (Figure 3d), whereas the 20 upregulated genes were associated with bacteriocin-related functions (Figure 3e).

### 2.4. Effect of S-PRG Filler Eluate on S. mutans Survival in Acidic Environments and Osmotic Pressure on Bacteria

**Figure 4:**
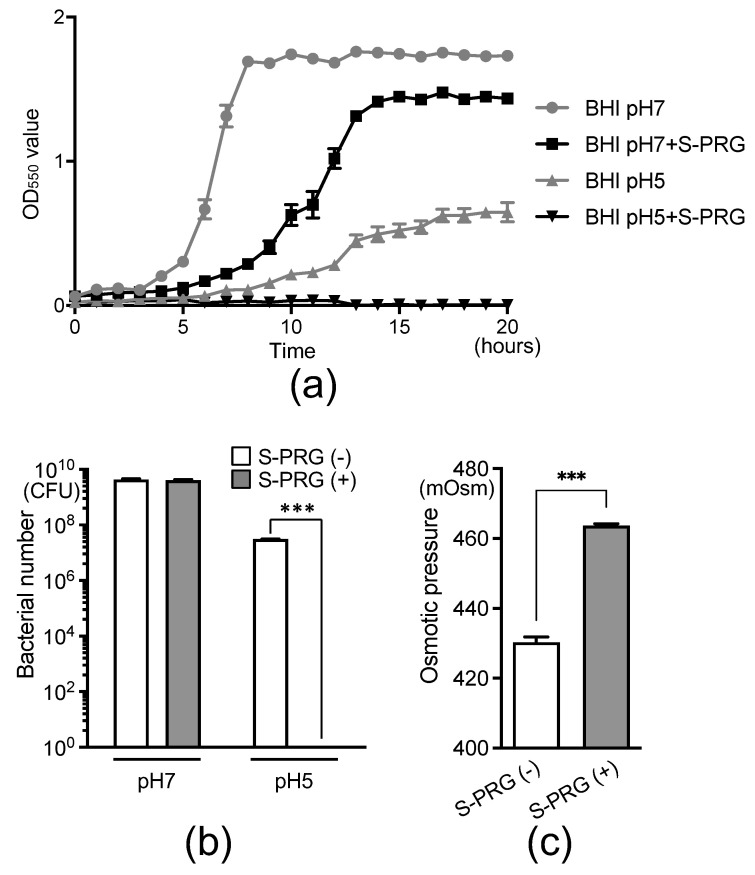
The effect of S-PRG filler eluate on the growth of *S. mutans* in the presence of sucrose and on the osmotic pressure of the bacterial culture. (**a**) Growth curves of *S. mutans* at different pH levels in the presence of 1% sucrose. (**b**) Bacterial counts of *S. mutans* at 20 h from (**a**). (**c**) Osmotic pressure in *S. mutans* cultures. Data are presented as means ± standard deviations. *** *p* < 0.001 between groups.

Because the downregulated *S. mutans* genes in the presence of the S-PRG filler eluate are involved in IMP biosynthesis and metabolism, both of which are related to acid tolerance, we analyzed the growth of *S. mutans* under different pH conditions. We monitored the growth of *S. mutans* with and without the S-PRG filler eluate at pH 7.0 and pH 5.0 by measuring the optical density at the 550 nm (OD_550_) value over time using GENESYS 30 (Thermo Fisher Scientific, Inc., Waltham, MA, USA) (Figure 4a). In the pH 7.0 suspension, bacterial growth was delayed in the presence of the S-PRG filler eluate compared with the absence of the S-PRG filler eluate. At pH 5.0, bacterial growth was inhibited in the absence of the S-PRG filler eluate compared with pH 7.0, and no *S. mutans* growth was observed in the presence of the S-PRG filler eluate for 20 h. Furthermore, 20 h after the start of growth, there was no significant difference in the number of *S. mutans* cells in the presence or absence of the S-PRG filler eluate at pH 7.0 (Figure 4b). However, at pH 5.0, the number of *S. mutans* cells was lower in the absence of the S-PRG filler eluate than at pH 7.0, and no *S. mutans* cells were detected in the presence of the S-PRG filler eluate.

Considering the upregulation of opuAa and opuAb, *S. mutans* genes related to osmotic stress, in the presence of the S-PRG filler eluate, we measured the osmotic pressure of the *S. mutans* solution. The osmotic pressure was significantly higher in the presence of the S-PRG filler eluate than in its absence (*p* < 0.001) (Figure 4c).

## 3. Discussion

This is the first study to comprehensively investigate the inhibitory effect of the S-PRG filler eluate on the presence of sucrose. Our research focused on the concentration of ions surrounding *S. mutans*, the comprehensive analysis of bacterial gene expression, and the associated effects on bacterial virulence. We found that several ions released from the S-PRG filler were present around *S. mutans*, and the S-PRG filler eluate effectively inhibited *S. mutans* by downregulating or upregulating gene expression.

The S-PRG filler is a type of bioactive functional glass that releases six ions and has a distinct ion release profile compared with glass ionomer cement [11]. The concentration of released ions differs between the S-PRG filler and conventional glass ionomer cement because fluoroboroaluminosilicate glass is used as the raw material for the S-PRG filler [11,14]. Fluoroboroaluminosilicate glass is completely different in composition from fluoroaluminosilicate glass, the main component of conventional glass ionomer cement, and it does not contain elements such as calcium and phosphorus. The concentrations of the six ions released into water from the S-PRG filler, particularly BO_3_^3−^, Sr^2+^, and F^−^, are relatively high, far exceeding those released from the unreacted filler of conventional glass-ionomer cement [11]. Miki et al. (2016) reported that BO_3_^3−^, Al^3+^, SiO_3_^2−^, and F^−^ released from the S-PRG filler could inhibit the growth of *S. mutans* [22]. However, the extent to which each ion is present around *S. mutans* in the presence of the S-PRG filler eluate is unknown. Our results showed that BO_3_^3−^, Al^3+^, Sr^2+^, and F^−^ were prominently detected in the presence of the S-PRG filler eluate, and their concentrations were further increased in the presence of sucrose. The inhibitory effect of the S-PRG filler on *S. mutans* is likely primarily attributed to these four ions and may be enhanced in the presence of sucrose, a factor that increases the risk of dental caries.

In dental materials containing an S-PRG filler, the S-PRG filler on the material’s surface initially releases a large concentration of ions, and then ions are gradually released from the entire material. In terms of clinical and dental material, evaluating the long-term effects of the S-PRG filler is essential. Meanwhile, in the presence of dental materials containing several percent to several tens of percent of the S-PRG filler, *S. mutans* that lose their ability to grow within a few minutes to a few hours do not recover their ability even after one month [12]. Therefore, this study focused on the short-term inhibitory effect of ions derived from the S-PRG filler on *S. mutans*.

We demonstrated the effect of using a supernatant that does not contain the S-PRG filler itself. This suggests that ions released from the S-PRG filler can inhibit *S. mutans* in the presence of sucrose, even if the S-PRG filler is not in direct contact with the tooth. In fact, ions released from the S-PRG filler in the composite resin pass through the adhesive layer and reach the tooth structure [23]. Ions derived from the S-PRG filler have also been detected in saliva [24]. In this study, we used the S-PRG filler eluate adjusted to a concentration of several percent to several tens of percent, which is the same as the S-PRG filler contained in the dental material. This methodology ensures the validity of our findings, as the ions may have an effect in areas that are not even in contact with the dental material.

Biofilm formation is a critical process in the development of dental caries. Biofilms can produce excessive acids in adverse conditions, such as high sugar intake and poor oral hygiene [25]. A prolonged low pH within the biofilm increases the proportion of acidogenic and aciduric microbes, disrupting the ecological balance and resulting in the development of dental caries [26]. Our study showed that the S-PRG filler eluate significantly reduced glucan formation and increased pH. Imazato et al. (2023) similarly reported that the S-PRG filler eluate inhibited glucan formation by *S. mutans* in the presence of sucrose, resulting in sparse biofilms with a depleted extracellular matrix [11]. These findings suggest that the S-PRG filler inhibits biofilm formation by *S. mutans* while suppressing the growth of acidogenic and aciduric microbes in dental plaque due to an elevated pH, thereby exerting a cariostatic effect.

In accordance with a previous microarray analysis of gene expression changes in *S. mutans* in the absence of sucrose [20], we used S-PRG eluates at concentrations of 6.3%, 12.5%, and 25% for the microarray analysis. Our microarray analysis identified 63 downregulated genes in the presence of the S-PRG filler eluate. STRING analysis revealed an association among these genes, including purC, purF, purL, purM, and purN, which are involved in purine biosynthesis [27]. Crowley et al. (1997) reported that the deletion of the fhs gene, involved in purine synthesis, rendered *S. mutans* significantly more sensitive to acid, highlighting the importance of purines in acid tolerance [28]. Additionally, GO enrichment analysis revealed that these downregulated genes function in IMP biosynthesis and metabolism, processes involved in the acid resistance of *S. mutans* [29]. Based on these findings, we hypothesized that the downregulation of these genes could affect acid tolerance; we investigated the growth of *S. mutans* at different pH values.

Most microorganisms within biofilms cannot survive when the local pH in the dental plaque microenvironment decreases to 5.0. However, *S. mutans* can dynamically adapt to acid stress (i.e., it displays aciduricity) [30], and acid tolerance plays a crucial role in its survival. In the presence of the S-PRG filler eluate, the growth of *S. mutans* was inhibited at pH 7.0, and no growth was observed at pH 5.0. These results indicate that the S-PRG filler eluate impairs the acid stress response of *S. mutans* by downregulating genes involved in acid tolerance. Welin-Neilands et al. (2007) demonstrated that fluoride inhibits the ability of *S. mutans* cells to induce an acid resistance response [31]. Therefore, ions released from the S-PRG filler eluate, including F^−^, may contribute to the altered acid resistance of *S. mutans*.

Microarray analysis revealed a strong association between the opuAa and opuAb operons among the 20 upregulated *S. mutans* genes in the presence of the S-PRG filler eluate. These genes respond to osmotic stress conditions in *Chromohalobacter salexigens* ANJ207 [32]. The oral environment is affected by osmolarity due to carbohydrates and salts, and oral bacteria can experience significant osmotic changes and must adapt to this stress [33]. Abranches et al. (2006) reported that opcA and opuAa, located in distinct operon-like arrangements, and Smu.2115, which flanks opcA, participate in the osmotic stress response of *S. mutans* [33]. Our results confirm that the S-PRG filler eluate caused high osmotic stress in *S. mutans*. Osmotic stress is known to induce the production of antibiotics and bacteriocins in bacteria [34]. This phenomenon may explain why the 20 upregulated genes were associated with bacteriocin-related functions in GO enrichment analysis.

S-PRG filler is incorporated into many dental products, such as composite resins, adhesives, cement, fissure sealants, toothbrushes, and polishing pastes [11]. The components contained in these materials affect the number of ions released by the S-PRG filler. In addition, when the material contains water or a component with a high affinity for water, the number of ions released from the S-PRG filler increases, while hydrophobic components hinder the ion release. It has been shown that many dental materials containing S-PRG filler inhibit the biofilm formation by *S. mutans*, but the effect varies depending on the component. In the future, the challenge will be to develop new materials that do not inhibit ion release.

**Figure 5:**
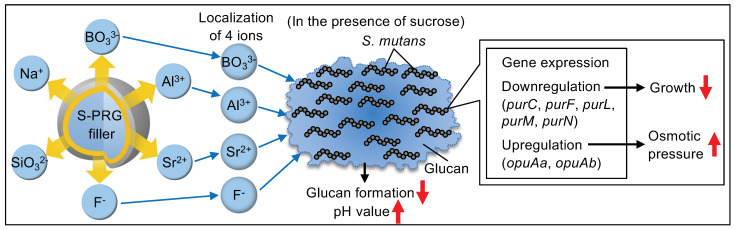
Schematic representation of the inhibitory effects of S-PRG filler eluates on *Streptococcus mutans* in the presence of sucrose.

Figure 5 summarizes our research findings. Four of the six types of ions released from the S-PRG filler are present around the glucan formed by *S. mutans* in the presence of sucrose. These ions inhibit glucan synthesis and increase the pH from acidic conditions. Additionally, S-PRG filler eluates decreased the expression of genes related to the biosynthetic processes of *S. mutans*, preventing bacterial growth at a low pH. Furthermore, the S-PRG filler eluate increased the expression of genes related to osmotic stress, raising osmotic pressure within the bacterial culture. These results suggest that the S-PRG filler eluate strongly inhibits the cariogenic activity of *S. mutans* in the presence of sucrose.

## 4. Materials and Methods

### 4.1. Preparation of S-PRG Filler

The method for preparing the bioactive functional glass S-PRG filler was described previously [14]. Briefly, fluoroboroaluminosilicate glass was heated to melt and then dried to obtain a glass frit consisting of 21.6% of SiO_2_, 21.6% of Al_2_O_3_, 16.6% of B_2_O_3_, 27.2% of SrO, 2.6% of Na_2_O, and 10.4% of F. The glass frit was wet-ground to obtain filler particles. The resulting glass slurry was mixed with polysiloxane, heat-treated, and crushed to obtain surface-treated glass filler. The surface-treated glass filler was sprayed with a polyacrylic acid aqueous solution while stirring and heat-treated to obtain the S-PRG filler.

### 4.2. Preparation of S-PRG Filler Eluate

The S-PRG filler eluate was prepared as previously described [20]. Briefly, the S-PRG filler was mixed with an equal volume of distilled water and gently agitated at room temperature for 24 h. The solution was then centrifuged at 3000× *g* at 23 °C for 6 h to separate the filler from the liquid. The supernatant was filtered (0.45 μm pore size) to remove any remaining insoluble material, and the resulting filtrate was used as the stock solution of the S-PRG filler eluate. The ion concentrations of the S-PRG eluate were as follows: Al^3+^ = 19.6 ppm, BO_3_^3−^ = 1656.5 ppm, Na^+^ = 618.5 ppm, SiO_3_^2−^ = 13.9 ppm, Sr^2+^ = 126.8 ppm, and F^−^ = 141.0 ppm [20]. Considering that the concentration of the S-PRG filler contained in dental materials is mainly several percent to several tens of percent [11,19,35], dilutions of the S-PRG eluate were used in each experiment.

### 4.3. Bacterial Strain and Growth Conditions

The *S. mutans* strain MT8148 was obtained from the stock culture collection in our laboratory [20]. The strain was cultured in Brain Heart Infusion (BHI) broth at 37 °C for 18 h, then adjusted in BHI broth to an OD_550_ of 1.0 (equivalent to 1 × 10^9^ colony-forming units [CFUs]/mL). The resulting suspensions were diluted to a final concentration of 1.0 × 10^7^ CFUs/mL, with or without 1% sucrose. These bacterial dilutions, with or without the 25% S-PRG filler eluate, were used in subsequent experiments.

### 4.4. Measurement of Ion Concentrations Surrounding S. mutans

A total of 10 mL of bacterial solution containing 1.0 × 10^7^ CFUs/mL of *S. mutans* and the 25% S-PRG eluate was cultured at 37 °C for 24 h to react *S. mutans* with S-PRG eluate. Then, the following preparations were immediately performed to measure the ion concentrations surrounding *S. mutans*. The S-PRG eluate remaining in the supernatant was then washed with phosphate-buffered saline (PBS) and removed by centrifugation. After the addition of PBS (10 mL), the bacterial pellet was sonicated to disrupt and separate the bacteria and the ions present around the bacteria, and the supernatant was collected by centrifugation. The concentrations of five ions (Al^3+^, BO_3_^3−^, Na^+^, SiO_3_^2−^, and Sr^2+^) associated with the S-PRG eluate in the supernatant (10 mL) were measured using an inductively coupled plasma atomic emission spectrometer (ICPS-8000, Shimadzu Corporation, Kyoto, Japan) used in previous studies [36,37]. The concentration of F^−^ was measured using an F^−^-selective electrode (model 9609BNWP, Orion Research Inc., Beverly, MA, USA) and an ion-selective electrode meter (model 720A, Orion Research Inc.).

### 4.5. Electron Microscopy Observations

Electron microscopy was performed as previously described [38]. Briefly, each bacterial sample was washed and fixed with 2% glutaraldehyde in PBS. After dehydration, the bacterial cells were embedded in epoxy resin (Epon, Nisshin-EM, Tokyo, Japan), cut into ultrathin sections, and observed by TEM.

### 4.6. Glucan Quantification

Glucan was quantified using a previously described method with minor modifications [39]. *S. mutans* (1.0 × 10^7^ CFUs/mL) was cultured at 37 °C for 24 h with and without 1% sucrose and the 25% S-PRG eluate. The bacterial solution was washed with PBS, and the supernatant was removed after centrifugation at 1000× *g* for 10 min. The pellet was then resuspended in 1 mL of 0.5 M NaOH, sonicated, and centrifuged. The total sugar content in the supernatant was determined using the phenol-sulfuric acid method, and the glucan amount was expressed in glucose equivalents.

### 4.7. DNA Microarray Analysis

To identify *S. mutans* genes affected by S-PRG eluate, we performed a systematic analysis of gene expression changes using DNA microarrays, as previously described [20]. Briefly, *S. mutans* MT8148 (1.0 × 10^7^ CFUs/mL) was cultured in BHI broth with varying concentrations of S-PRG eluate at 37 °C for 18 h. Amino-allyl-amplified RNA was generated from total RNA using the Amino-allyl MessageAmp aRNA kit (Ambion, Inc., Austin, TX, USA). RNA purity, concentration, and quality were confirmed using an Agilent 2200 TapeStation (Agilent Technologies, Inc., Santa Clara, CA, USA). RNA purity and quality were assessed by nucleic acid absorbance ratios (A_260_/A_230_ and A_260_/A_280_), which were greater than 2.0 for all samples. The RNA concentrations ranged from 51.6 to 60.0 ng/μL. All samples were adjusted to a minimum concentration of 50.0 ng/μL before microarray analysis. 

Microarray assays were conducted using Agilent Technologies products in accordance with the manufacturer’s protocols. Briefly, Cy3-labeled complementary RNA (cRNA) was prepared using the Low Input Quick Amp Labeling Kit, One-Color (Agilent Technologies). The Cy3-labeled cRNA was hybridized with the complete genome of *S. mutans* UA159 assembled with the Agilent Expression Array kit. After samples had been washed with the Gene Expression Wash Buffers Pack (Agilent Technologies), hybridization images were analyzed using an Agilent Microarray Scanner (G2565CA) (Agilent Technologies). Quantitative data were obtained using Agilent Feature Extraction Software 9.5.1.1 (Agilent Technologies), and background signal intensity corrections were performed as previously described [40]. 

Altered genes were identified using three different comparisons for each *S. mutans* strain, focusing on the following S-PRG eluate concentrations: 0% versus 6.3%, 0% versus 12.5%, and 0% versus 25.0%. We selected genes with a log2 ratio change greater than 1.0 in the presence or absence of the indicated S-PRG filler eluate concentrations.

### 4.8. Bioinformatic Analyses

Bioinformatic analyses were performed using the previously described method, with minor modifications [41]. We used genes identified in the DNA microarray analysis to establish PPI networks based on the StringApp11.5 (Search Tool for the Retrieval of Interacting Genes/Proteins) online database (https://string-db.org/, accessed on 3 July 2024). Then, the strongest modules in the PPI networks were visualized. GO enrichment analysis was performed using ShinyGO 0.80 online resources (http://bioinformatics.sdstate.edu/go/, accessed on 3 July 2024). A false discovery rate-corrected *p*-value cutoff of 0.05 was used to determine genes for inclusion in the GO enrichment analysis.

### 4.9. Bacterial Growth Assay

The bacterial growth assay was performed as previously described, with minor modifications [41,42]. Cultured bacteria were inoculated into BHI broth adjusted to pH 7.0 or pH 5.0, with or without the 25% S-PRG filler eluate, at a final concentration of 1.0 × 10^7^ CFUs/mL. Bacterial growth was monitored at 37 °C for 20 h by measuring the OD_550_. After 20 h of incubation, the bacteria were spread onto MSB agar plates and incubated anaerobically at 37 °C for 48 h. Colony counts were then performed.

### 4.10. Statistical Analysis

Statistical analyses were conducted using GraphPad Prism 9 software (GraphPad Software Inc., La Jolla, CA, USA). The Wilcoxon rank-sum test was used to compare groups based on the presence or absence of sucrose or the S-PRG filler eluate. Differences were considered statistically significant at *p* < 0.05.

## 5. Conclusions

Several ions released from the S-PRG filler were present around *S. mutans* in the presence of sucrose. These ions inhibited glucan synthesis by *S. mutans* and increased the pH from acidic conditions. Furthermore, the S-PRG filler eluate prevented bacterial growth at a low pH and increased osmotic pressure within the bacterial culture by down- or upregulating the gene expression of *S. mutans*. These results suggest that the S-PRG filler eluate effectively suppresses the cariogenicity of *S. mutans* in the presence of sucrose.

## Data Availability

The data are available from the corresponding author upon reasonable request.
